# Effect of Dentifrice Ingredients on Volume and Vitality of a Simulated Periodontal Multispecies Biofilm

**DOI:** 10.3390/dj12050141

**Published:** 2024-05-13

**Authors:** Jelena Karacic, Moritz Ruf, Johannes Herzog, Monika Astasov-Frauenhoffer, Philipp Sahrmann

**Affiliations:** 1 Department of Periodontology, Endodontology and Cariology, University Center for Dental Medicine Basel UZB, University of Basel, Mattenstrasse 40, CH-4058 Basel, Switzerland; moritz.ruf@unibas.ch (M.R.); philipp.sahrmann@unibas.ch (P.S.); 2 Department Research, University Center for Dental Medicine Basel UZB, University of Basel, Mattenstrasse 40, CH-4058 Basel, Switzerland; m.astasov-frauenhoffer@unibas.ch

**Keywords:** step 1 periodontal therapy, oral hygiene, biofilm removal, preventive dentistry, antibacterial agents

## Abstract

The aim of this in vitro study was to investigate the effect of different toothpaste ingredients on biofilm volume and vitality in an established non-contact biofilm removal model. A multi-species biofilm comprising *Porphyromonas gingivalis*, *Streptococcus sanguinis*, and *Fusobacterium nucleatum* was grown on protein-coated titanium disks. Six disks per group were exposed to 4 seconds non-contact brushing using a sonic toothbrush. Four groups assessed slurries containing different ingredients, i.e., dexpanthenol (DP), peppermint oil (PO), cocamidopropyl betaine (CB), and sodium hydroxide (NaOH), one positive control group with the slurry of a toothpaste (POS), and a negative control group with physiological saline (NEG). Biofilm volume and vitality were measured using live-dead staining and confocal laser scanning microscopy. Statistical analysis comprised descriptive statistics and inter-group differences. In the test groups, lowest vitality and volume were found for CB (50.2 ± 11.9%) and PO (3.6 × 10^5^ ± 1.8 × 10^5^ µm^3^), respectively. Significant differences regarding biofilm vitality were found comparing CB and PO (*p* = 0.033), CB and NEG (*p* = 0.014), NaOH and NEG (*p* = 0.033), and POS and NEG (*p* = 0.037). However, no significant inter-group differences for biofilm volume were observed. These findings suggest that CB as a toothpaste ingredient had a considerable impact on biofilm vitality even in a non-contact brushing setting, while no considerable impact on biofilm volume was found.

## 1. Introduction

Biofilm-caused oral diseases are widespread and a global challenge despite the extensive knowledge about its considerable impact on public health [1]. Among these diseases, periodontal diseases do not only severely harm the function and health of teeth and attachment apparatus, they also have a noxious effect on general health, negatively affecting the course of comorbidities like diabetes mellitus and cardiovascular events. They have also been shown to lower quality of life due to body image issues, social isolation, anxiety, and functional limitations [1,2]. 

The primary prevention of periodontal diseases is the accepted gold standard in combating them, as they have been proven to be largely avoidable [3]. The first step of periodontal therapy comprises the change of patient habits [4]. The primary focus is thereby set on oral hygiene and the implementation of professional mechanical plaque removal (PMPR), as outlined in recent guidelines [4]. While the use of antiseptic rinses has a minor effect on dental biofilms, mechanical debridement as performed by patient-administered brushing is considered the gold standard for effective biofilm control [5]. 

Achieving complete mechanical biofilm removal by oral hygiene measures remains challenging, particularly in difficult-to-access regions such as interproximal areas [6]. Sonic toothbrushes have been reported to remove biofilms without bristle contact by fluid flow causing hydrodynamic forces [7], thermodynamic surface tension forces [8], energy transfer by bristles vibration [9], and acoustic energy due to oscillation [10]. Schmidt et al. established an in vitro model assessing the optimal brushing distance and brushing time for different tooth surfaces and evaluating the overall efficacy of side-to-side toothbrushes, which allowed for the specific assessment of the brushing effect in areas that are not reached by the bristles [11]. In that set-up, sandblasted, large grit, acid-etched (SLA) titanium disks were used, promoting a highly standardized substrate for initial bacterial colonization in a dynamic flow chamber system, mimicking the saliva flow and applying shear forces that are important for the mechanical characteristics of biofilms. Given a total brushing time of 2 to 3 min, exposures of up to 6 seconds per test surface have been considered clinically realistic [12,13]. Other in vitro studies using comparable models have assessed the impact of powered toothbrushes with diverse modes of action on non-contact brushing [9,14,15,16]. In contrast to models with monospecies biofilms employed in prior studies [16,17,18], the in vitro biofilm formation in this study utilized a multispecies biofilm. Most models were set up with a toothbrush angulation of 90° toward the biofilm [9,16,18]. Thus, the interproximal toothbrush apparatus utilized by the study group of Hope et al. exhibited the closest resemblance to our experimental setup [15,19].

Chemical agents as ingredients of dentifrices, gels, mouth rinses, and others may play a supplementary purpose in biofilm removal. Antiseptic adjuncts in toothpastes claim the potential to enhance biofilm removal, supplementing mechanical cleaning with toothbrushes. Several substances have already been evaluated as active agents in dentifrices. In this study, we initially tested the solubility of ingredients found in a common toothpaste. Subsequently, soluble ingredients were selected based on their possible antibacterial properties. Dexpanthenol is an active ingredient of many toothpastes with anti-inflammatory effects on dental biofilm [20]. Furthermore, a recent study from Madrazo-Jiménez et al. found improved wound healing after wisdom tooth extraction with the application of gel containing dexpanthenol [21]. Similar results were found with a reduction of postoperative pain and signs of inflammation in a randomized and placebo-controlled clinical trial [22]. Another in vitro study showed that a dexpanthenol-containing ointment had a favorable impact on structures critical to antimicrobial barrier function and had anti-inflammatory effects after photon irradiation [23].

Peppermint oil is often used as a flavoring agent in dentifrices, but has also been shown to reduce plaque formation, decrease salivary *S. mutans* counts, and improve the salivary buffer capacity [24,25]. Antibacterial and antifungal properties have also been described [26]. Its inhibitory effect on oral bacteria biofilm formation was demonstrated both in vitro and in vivo [27]. 

Cocamidopropyl betaine (CB) is a detergent which is typically used in concentrations of 1–2% in dentifrices. It reduces the irritating effect of sodium dodecyl sulfate (SDS), another detergent widely used in dentifrices, and relieves dry mouth symptoms [28,29]. Compared to SDS, CB is suggested for patients with a high risk of caries due to its greater effect on remineralization [30].

Sodium hydroxide (NaOH) is commonly used in dentifrices as an ingredient to adjust the pH value and neutralize other acidic ingredients. There are few studies assessing the antibacterial effect of NaOH on periodontal pathogens. In a study by Allan et al. on the antiseptic effects of bioglass, NaOH was found to reduce 97.2% of *S. sanguis* already at a very low concentration (6.31 × 10^−6^%) in a supernate [31]. On the other hand, in another laboratory study, pre-exposure of *S. mutans* to 0.1 M NaOH did not alter the functionality of those bacteria in terms of their ability for 18F accumulation [32]. 

Until today, the exact and comprehensive mechanisms of action of these substances are not yet understood, nor are their optimal dosage and combination for effective biofilm removal in dentifrices. These knowledge gaps underscore the ongoing challenge in fully elucidating the intricacies of their action. Taken together, chemical agents might either reduce biofilm volume quantitatively, or they might affect its functionality in terms of a bactericidal effect, thereby killing viable cells within the biofilm structure without necessarily removing them. Clarifying these mechanisms and their implications remains an essential area for further research. Therefore, the aim of the present study was to investigate the effects on biofilm volume and vitality of different ingredients in a common toothpaste in an established non-contact brushing model. It was the null hypothesis of this experiment that neither biofilm volume nor vitality would be affected by the different test substances.

## 2. Materials and Methods

### 2.1. Sample Size Calculation

An a priori sample size calculation was performed, based on data stated in a former study [11,12,13,33]. In summary, considering a minimal benefit of an additional 69% of biofilm removal with a significance level set at 5% and intending for a test power of 80%, a least amount of 4 disks per group was defined as the required minimum. Attributing for the probability of slight variances in biofilm volume and vitality outcomes, a total of six specimens per group was defined to accommodate a sensible safety limit.

### 2.2. Biofilm Preparation

A multispecies biofilm was used based on a previously described protocol [11,12,13,33,34]. Concisely, applying a flow chamber system together with a static biofilm growth model, *Fusobacterium nucleatum* ATCC 10953, *Streptococcus sanguinis* DSM 20068, and *Porphyromonas gingivalis* DSM 20709 were utilized for biofilm formation [35,36,37,38]. Sterile disks of commercially pure titanium (Grade 2, ASTM F-67), with a diameter of 5 mm and 1 mm of thickness, with a SLA surface (Institut Straumann AG, Basel, Switzerland) worked as substrates after incubation for 15 minutes in a serum/saliva mixture (ethical approval EKBB 295/08, by Research Ethics Committee of the University of Basel, Switzerland). This facilitated protein pellicle formation. Fasting-simulated saliva from healthy volunteers and pooled serum (Blutspendezentrum, Basel, Switzerland) was mixed based on the established protocol from Hauser–Gerspach et al. [38]. The protein-coated substrates were inserted into an anaerobic flow chamber, as previously described in detail [36,37,38,39]. Anaerobic conditions (MACS MG; Don Whitley Scientific Ltd., West Yorkshire, UK) with an atmosphere of 80% N_2_, 10% H_2_, and 10% CO_2_ at 37 °C were created for circulation of the bacterial suspension at 0.8 mL/min for 72 h. Every 24 h, the suspension was renewed. Disks were placed in wells, following removal from the anaerobic flow chamber, of a 12-well plate loaded with a compound of thioglycolate (bioMerieux SA, Geneva, Switzerland) enhanced with 0.5 μg/mL menadione (VWR International, Dietikon, Switzerland) and 5 μg/mL hemin (Fluka, Buchs, Switzerland) along with simulated body fluid [40] (1:1) supplemented with 0.2% glucose. After anaerobic incubation in a single well at 37 °C for 18 h of each biofilm-coated substrate, the total biofilm growth time was 90 h.

### 2.3. Exposition to Non-Contact Brushing

A sonic-driven side-to-side toothbrush (Philips^®^ Sonicare FlexCare HX6902/02 Philips GmbH, Hamburg, Germany; “Clean-Modus”, 31,000 strokes per minute) was selected following a previously published experimental model [12,13,33]. Accordingly, the toothbrush and biofilm-coated disks were placed in an adjustable and individually manufactured experimental set-up for non-contact brushing with a toothbrush angulation of 45° and with the bristles “just not touching” the disk at a distance of 0 mm. The sonic activation of the toothbrush was generally set at four seconds.

For each experiment, 3 g of toothpaste and the respective quantity analog of the specific ingredients were used. Therefore, the individual substances were tested in a pre-study to determine whether the respective amounts could be homogeneously mixed in NaCl. Single ingredients of a commercially available dentifrice (paro^®^amin, Esro AG, Paroswiss, Kilchberg, Switzerland) were chosen as test groups. In order to achieve the respective concentrations of the ingredients used in the test groups slurries within the dentifrice, their proportions were determined through calculations using data provided by their manufacturer. The individual amounts of the individual ingredients were doubled (6 g) with 20 mL of 0.9% NaCl to form a slurry. For the positive control group (POS), 6 g of the original toothpaste was mixed with 20 mL NaCl. Homogenous slurries were created for the groups POS, dexpanthenol (DP, 20 mL slurry with NaCl and 600 µL DP), peppermint oil (PO, 20 mL slurry with NaCl and 900 µL PO), cocamidopropyl betaine (CB, 20 mL slurry with NaCl and 1200 µL), and CB and sodium hydroxide (NaOH, 20 mL slurry with NaCl and 60 µL NaOH) by vortexing in a magnetic stirrer at 400 rpm for 10 min and additionally at 500 rpm again for 10 min directly before usage. Of the so-formed slurry, 10 mL was applied into the container of the interdental model setup. For testing, the biofilm-coated disks and the toothbrush bristles were submerged in the solutions. The ingredients for the test group DP, PO, CB, and NaOH were supplied by Esro AG (Paroswiss, Kilchberg, Switzerland).

After treatment, the samples were stored in a toothpaste inactivation solution (1 g tryptone and 8.5 g NaCl dissolved in 1 L H_2_O, to this 1 g L-histidine, 5 g Na_2_S_2_O_3_, 3 g lecithin, and 94 mL Tween-80 was added; all Sigma-Aldrich, Buchs, Switzerland) in order to stop the reaction [41].

### 2.4. Staining and Microscopical Analysis

Following a prior described protocol [34], the disks were prepared for live/dead staining (Live/dead BacLightTM bacterial Viability Kit; MoBiTec, Luzern, Switzerland) and analyzed under a Leica SP8 inverted confocal laser scanning microscope (CLSM; Leica Microsystems AG, Heerbrugg, Switzerland). The application of two fluorescent dyes enabled the distinction between vital and dead cells. In brief, propidium iodide exclusively marks cells with damaged membranes, a finding solely in dead cells, and Syto9 stains all cells. Microscopic fluorescence examination using lasers at 488 nm (FITC filter) and 552 nm (PI filter) revealed first all cells and then those which were stained by both dyes, i.e., the cells considered ‘‘dead’’. The proportion of volumes for alive and dead cells in the entire biofilms were calculated using volumetric analyses with Imaris software 9.5.0 (Oxford Instruments plc, Abingdon, UK). For the analysis of biofilm volume, the mean volumes of the biofilms (comprising both live and dead cells) on the exposed substrates were combined by summation.

Thirty-six biofilm-coated titanium disks, each containing six discs in a total of six independent flow chamber experiments, were utilized to produce one for the exposure to each of the different ingredients. Analysis was conducted on three randomly chosen microscopic fields located near the center of each disk. Means and standard deviations of the biofilm volumes were determined. Medians, interquartile ranges (IQRs), and full range of values were presented as boxplots for each experimental setting. Normal distribution analysis was performed by Kolmogorov–Smirnov tests. Finding skewed distributions of the standard deviations, the non-parametric Kruskal–Wallis test was employed for inter-group analysis. The significance level was set at α = 0.05. All computations were carried out utilizing SPSS^®^ software (SPSS^®^ Statistics 25.0.0.0; SPSS Inc., Chicago, IL, USA).

## 3. Results

**Figure 1:**
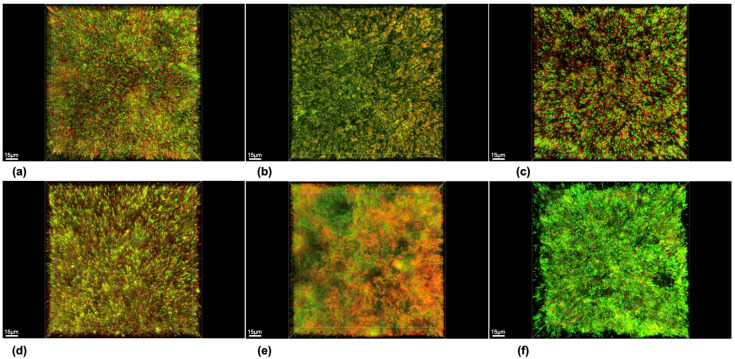
Representative images by confocal laser scanning microscope (CLSM). Red indicates cells considered to be dead and green alive cells. (**a**) Dexpanthenol (DP), (**b**) peppermint oil (PO), (**c**) cocamidopropyl betaine (CB), (**d**) sodium hydroxide (NaOH), (**e**) positive control group with original toothpaste (POS), (**f**) negative control group with NaCl (NEG).

The representative CLSM images of the biofilm in the negative control group showed a well-established biofilm growth with a high amount of live cells (Figure 1) with a mean volume of 6.6 × 10^5^ µm^3^ and biofilm vitality of 68.24% (Table 1).

**Figure 2:**
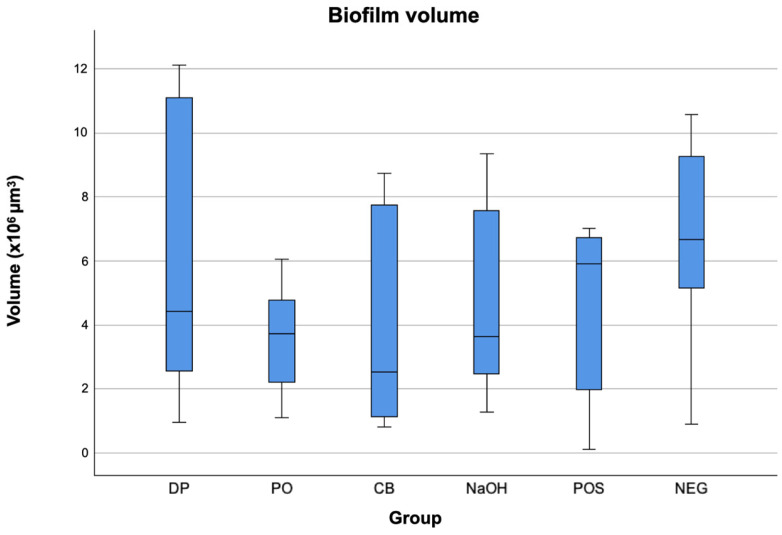
Overall biofilm volume (µm) after non-contact brushing for 4 seconds with a brushing distance (artificial tooth-to-bristle) of 0 mm, a toothbrush angulation of 45° and a simulated interdental space width of 1 mm: mean volumes from the independent experiments (*n* = 36) for group dexpanthenol (DP), peppermint oil (PO), cocamidopropyl betaine (CB), sodium hydroxide (NaOH), positive control group with original toothpaste (POS), and negative control group with NaCl (NEG). The boxplots indicate interquartile ranges (IQRs), the horizontal line indicate the medians, and the whiskers show the full range of values. No statistically significant inter-group differences were found (Kruskal–Wallis test with significance level of 0.05).

**Figure 3:**
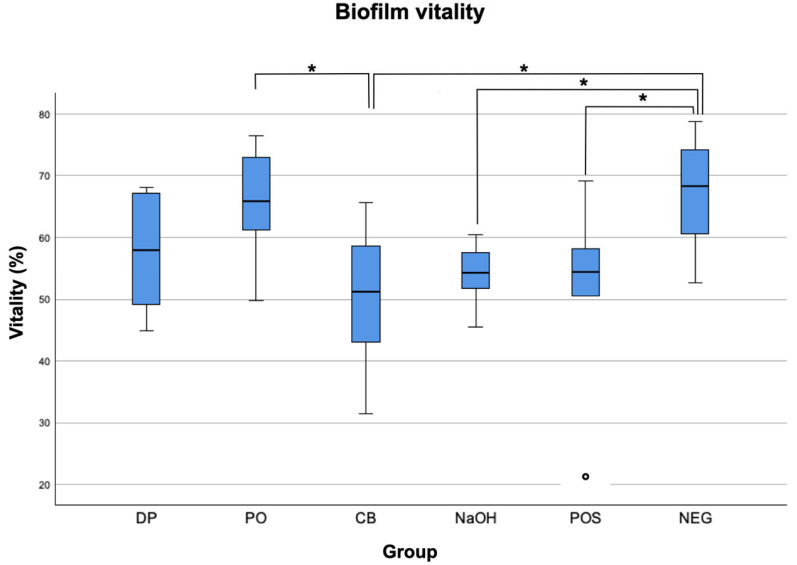
Overall biofilm vitality (%) after non-contact brushing for 4 seconds with a brushing distance (artificial tooth-to-bristle) of 0 mm, a toothbrush angulation of 45° and a simulated interdental space width of 1 mm: mean volumes from the independent experiments (*n* = 36) for group dexpanthenol (DP), peppermint oil (PO), cocamidopropyl betaine (CB), sodium hydroxide (NaOH), positive control group with original toothpaste (POS), and negative control group with NaCl (NEG). The boxplots indicate interquartile ranges (IQRs), the horizontal line indicate the medians, and the whiskers show the full range of values. The ° symbol represents an outlier greater than twice the median value. The statistically significant inter-group differences between CB and PO, CB and NEG, NaOH and NEG, and POS and NEG (Kruskal–Wallis test, *p* < 0.05) are indicated by *.

Lowest biofilm volume was observed for CB (2.5 × 10^5^ µm^3^), followed by PO (3.7 × 10^5^ µm^3^), DP (4.4 × 10^5^ µm3), POS (5.9 × 10^5^ µm^3^), and lastly NEG (6.6 × 10^5^ µm^3^). Lowest biofilm vitality was found in group CB with a proportion of 51.27%. Similar results were found for group POS (54.40%) and NaOH (54.35%). The highest remaining biofilm vitality was found in groups DP (58.00%), PO (65.91%), and NEG (68.24%). The mean volumes and vitalities of simulated interdental biofilms after brushing in different slurries are shown in Figure 2 and Figure 3.

Based on the Kolmogorov–Smirnov tests, standard deviations of the data set were not normally distributed, indicating a non-parametric Kruskal–Wallis test for inter-group comparisons.

Regarding biofilm volume, there were no significant differences between the groups.

As for biofilm vitality, there were significant differences between the negative control group NEG and the groups CB (*p* = 0.014), NaOH (*p* = 0.033), and POS (*p* = 0.037) (Table 2). Furthermore, the vitality of group CB showed a significant difference to group PO (*p* = 0.033).

## 4. Discussion

In general, there is limited evidence demonstrating that ingredients of a dentifrice may quantitatively and qualitatively affect oral biofilms. Thus, this study aimed to explore the effect of different ingredients of a commercially available toothpaste containing CB, NaOH, PO, and DP on biofilm volume and vitality. In an established in vitro setup, first described by Schmidt et al., we found that while a reduction of biofilm volume was not significantly influenced by the specific ingredients, vitality in the biofilms was affected by different substances [11]. Therefore, we found our null hypothesis partly rejected.

Oral biofilm removal is generally performed mechanically. Chemical removal with dentifrices does not seem to provide an additional effect on plaque removal compared to mechanical biofilm removal alone [42]. Today, the exception regarding additional plaque removal seems to be dentifrices with anti-inflammatory agents like triclosan or stannous fluoride [43].

Effective biofilm volume reduction or a change in the biofilm’s vitality is either possible by primarily antiseptic ingredients or by the use of surfactants. The latter might facilitate a more effective penetration of antiseptic agents into the biofilm by lowering the surface tension, or just reduce its volume by micelle-based detachment. This missing effect could be due to the low non-contact forces.

The lacking effect on biofilm volume by the ingredients may be due to the strong adhesion and cohesion capability within this in vitro biofilm. This capability could exceed the tenside-like impact of the ingredients, particularly CB. The lacking effect shown in the in vitro experiment is unlikely to be stronger in the clinical situation, due to stronger biofilm cohesion as a result to higher shear forces, and the multi-species composition of biofilms in the oral cavity. The anatomical barrier of the gingival margin further protects subgingivally located periodontal biofilms from the access by both mechanical and chemical means.

Significant differences for biofilm vitality were found for CB and NaOH compared to the negative control group. However, NaOH is not present as a free agent; instead, it is bound within the formulation and serves to neutralize acidic compounds, thereby influencing the pH value of the toothpaste. Nonetheless, a comparison with other studies regarding their influence on biofilm volume and vitality is not possible due to lacking evidence.

The exception among the tested ingredients is PO, whose antimicrobial effect on *P. gingivalis* was established at 30–50 µg/mL in another in vitro study [44]. However, the present study, using 900 µL of PO, did demonstrate the lowest biofilm volume but not significantly. This disparity can be attributed to differences in outcome measures; while this study assessed biofilm volume and vitality, the other study focused on microbial growth inhibition.

These findings cannot be directly transferred into clinics due to the limitations of the in vitro set-up of the present experiment. Another limitation is the non-contact brushing modality. The hypothesized shear and hydrodynamic forces which result in non-contact brushing through transferring energy [8,17] could be hindered by toothpaste slurries. A possible cause is the influence of different liquid viscosities on fluid flow, bristle velocity, shear forces, and the formation of air bubbles [45].

Another limitation of the present study is that only selected active ingredients of the common dentifrice were investigated for their biofilm removal and vitality effect. The respective selection of assessed ingredients was based on two aspects: First, ingredients with a known or presumable effect on biofilm volume or vitality were chosen. Then, ingredients had to be completely dissoluble in NaCl in order to form a homogenous slurry, not allowing for example the test of methylparaben which was not soluble without the simultaneous use of ethanol.

The amount of the tested ingredients in the slurries was set based on previous studies by Difloe et al. working with 6 g dentifrice, therefore targeting the respective amounts of ingredients in such a toothpaste portion [46]. The dosage of the materials was calculated according the manufacturer instructions. To our knowledge there are no data regarding the contribution of different factors and the ideal dosage for highest non-contact biofilm removal. Furthermore, previous investigations focused mostly on caries protection [47], management of dentin hypersensitivity [48], impact on abrasiveness [49], influence on gingival health [50], and inhibition of plaque regrowth instead of plaque removal [51]. Thus, the impact of different dosages and possible interactions of active ingredients could improve the efficiency of biofilm removal for dentifrices. Finally, the use of titanium discs instead of enamel or dentin discs as a substrate is a difference to the situation in the oral cavity. The choice of titanium discs, however, was made due to the better standardization, and the comparability to the previously established set-up.

The findings of this study are in accordance with previous studies, which show that toothpaste slurries decrease the efficacy of biofilm reduction potential of non-contact toothbrushing only [46]. The dentifrice and its active ingredients seem to only have an effect on biofilm vitality, but not on biofilm volume reduction. This corroborates the EFP-3 guidelines recommendations of focusing on the mechanical removal of biofilm for preventing and treating periodontal diseases [4]. Mechanical biofilm removal remains to be the most effective way of reducing biofilm and cannot be adequately replaced by chemical ingredients in dentifrices. Nevertheless, it should be the aim of future studies to investigate the role of various toothbrush ingredients on biofilm reduction and vitality in order to assess the potential adjunctive effect to mechanical debridement.

## 5. Conclusions

Within the limitations of the in vitro study design, several conclusions were drawn. Firstly, none of the groups exhibited the capability to significantly reduce the biofilm volume. However, it was observed that both cocamidopropyl betaine and sodium hydroxide possessed the ability to reduce biofilm vitality. Despite this, the overall impact of the ingredients appeared to be minor. Consequently, it is evident that further research is necessary to elucidate which substances, at specific concentrations, might hold the potential to enhance non-contact biofilm removal. While these results are based on clinical testing, their direct implications may be limited. Nevertheless, they could prove valuable for shaping future studies.

## Figures and Tables

**Table 1:** Medians and interquartile ranges (IQRs) of biofilm volume (×10^5^ µm^3^) and biofilm vitality (%) values of the materials.

Group	Biofilm Volume (×10^5^ µm^3^)		Biofilm Vitality (%)	
	Medians	IQRs	Medians	IQRs
DP ^1^	4.4 ^a^	9.2	58.00 ^a^	19.37
PO ^2^	3.7 ^a^	3.2	65.91 ^ab^	15.51
CB ^3^	2.5 ^a^	6.9	51.27 ^ac^	20.22
NaOH ^4^	3.6 ^a^	5.8	54.35 ^abcd^	8.06
POS ^5^	5.9 ^a^	5.3	54.40 ^abcde^	17.63
NEG ^6^	6.6 ^a^	5.5	68.24 ^abf^	16.69

^1^ Dexpanthenol (DP), ^2^ peppermint oil (PO), ^3^ cocamidopropyl betaine (CB), ^4^ sodium hydroxide (NaOH), ^5^ positive control group with original toothpaste (POS), ^6^ negative control group with NaCl (NEG), ^abcdef^ within a row, means without a common superscript differ (*p* < 0.05).

**Table 2:** *p*-values for inter-group comparisons of biofilm vitality (Kruskal–Wallis test). Statistically significant values are given in bold letters.

	DP ^1^	PO ^2^	CB ^3^	NaOH ^4^	POS ^5^	NEG ^6^
DP		*p* = 0.171	*p* = 0.443	*p* = 0.661	*p* = 0.701	*p* = 0.089
PO	*p* = 0.171		*p* = 0.033	*p* = 0.071	*p* = 0.08	*p* = 0.742
CB	*p* = 0.443	*p* = 0.033		*p* = 0.742	*p* = 0.701	*p* = 0.014
NaOH	*p* = 0.661	*p* = 0.071	*p* = 0.742		*p* = 0.956	*p* = 0.033
POS	*p* = 0.701	*p* = 0.08	*p* = 0.701	*p* = 0.956		*p* = 0.037
NEG	*p* = 0.089	*p* = 0.742	*p* = 0.014	*p* = 0.033	*p* = 0.037	

^1^ Dexpanthenol (DP), ^2^ peppermint oil (PO), ^3^ cocamidopropyl betaine (CB), ^4^ sodium hydroxide (NaOH), ^5^ positive control group with original toothpaste (POS), ^6^ negative control group with NaCl (NEG).

## Data Availability

The original contributions presented in the study are included in the article; further inquiries can be directed to the corresponding author.
